# Characterization of GATA3 and Mammaglobin in breast tumors from African American women

**DOI:** 10.21203/rs.3.rs-2463961/v1

**Published:** 2023-01-25

**Authors:** Luisel J. Ricks-Santi, Kristianna Fredenburg, Moein Rajaei, Ashwin Esnakula, Tammey Naab, J. Tyson McDonald, Yasmine Kanaan

**Affiliations:** Department of Pharmacotherapy and Translational Research, College of Pharmacy, University of Florida, Gainesville, FL; Department of Pathology, College of Medicine, University of Florida, Gainesville, FL; Department of Pharmacotherapy and Translational Research, College of Pharmacy, University of Florida, Gainesville, FL; Department of Pathology, The Ohio State University Wexner Medical Center, Columbus, OH; Department of Pathology, Howard University Hospital, Washington, DC; Department of Radiation Medicine, Georgetown University School of Medicine, Washington D.C; Department of Microbiology, Howard University College of Medicine, Washington, DC

**Keywords:** breast cancer, GATA3, Mammaglobin, triple negative breast cancer, immuniohistochemistry

## Abstract

GATA3 and Mammaglobin are often used in the clinic to identify metastases of mammary origin due to their robust and diffuse expression in mammary tissue. However, the expression of these markers has not been well characterized in tumors from African American women. The goal of this study was to characterize and evaluate the expression of GATA3 and mammaglobin breast tumors from African American women and determine their association with clinicopathological outcomes including breast cancer subtypes. Tissue microarrays (TMAs) were constructed from well preserved, morphologically representative tumors in archived formalin-fixed, paraffin-embedded (FFPE) surgical blocks from 202 patients with primary invasive ductal carcinoma. Mammaglobin, and GATA3 expression was assessed using immunohistochemistry (IHC). Univariate analysis was carried out to determine the association between expression of GATA3, mammaglobin and clinicopathological characteristics. Kaplan-Meier estimates of overall survival and disease-free survival were also plotted and a log-rank test performed to compare estimates among groups. GATA3 expression showed statistically significant association with lower grade (p<0.001), ER-positivity (p<0.001), PR-positivity (p<0.001), and the luminal subtype (p<0.001). Mammaglobin expression was also significantly associated with lower grade (p=0.031), ER-positivity (p=0.007), and PR-positivity (p=0.022). There was no association with recurrence-free or overall survival. Our results confirm that GATA3 and mammaglobin demonstrate expression predominantly in luminal breast cancers from African American women. Markers with improved specificity and sensitivity are warranted given the high prevalence of triple negative breast cancer in the group.

## Background

Tumors originating from breast tissue can be identified through the use of mammary-specific markers such as mammaglobin and estrogen receptor (ER). The aforementioned markers also assist with the identification of tumors of unknown primaries and metastases[1][2–6]. Mammaglobin is generally positive in normal breast epithelium, as is the ER protein. However, given their frequent absence in breast cancer metastases and triple negative breast cancer (TNBC)[2–6], additional markers such as GATA Binding Protein 3 (GATA3) are being used to distinguish tumors originating from the breast. GATA3 is a transcription factor with a role in cell proliferation and differentiation of breast luminal epithelial cells. GATA3 and ER are closely associated and are involved in a positive cross-regulatory loop explaining the positive correlation between GATA3 and ER expression in breast cancers. Notably, GATA3 has been found to be more sensitive in detecting metastatic breast tumors in cytologic specimens[7] and several studies have also suggested a prognostic or predictive role for GATA3 expression[8–11]. GATA3 is expressed in breast and urothelial carcinomas while mammaglobin may be expressed in breast, salivary gland, and endometrial carcinomas.

However, GATA3 expression varies in breast cancer molecular subtypes. For example, GATA3 expression ranges from 93–100% in Luminal tumors, 59–94% in HER2 overexpressing tumors, and 20–44% in TNBCs[12–17]. It is well known that compared to Caucasian women, African American women are almost twice as likely to be diagnosed with TNBC (ER-negative, PR-negative, and HER2-negative)[18–22]. Therefore, GATA3 may have limited clinical utility in the population. Given the higher frequency of metastatic breast cancer and TNBC in the group, identifying improved diagnostic markers, as well markers that can identify the origin of the tumor is paramount for prognostication, determining treatment options, and deploying treatment options in a timely manner. The goal of this study was to characterize and evaluate the expression of GATA3 and mammaglobin in breast tumors from African American women and determine their association with clinicopathological outcomes including breast cancer subtypes. It is hypothesized that their expression will be reduced in TNBC and will be associated with prognostic indicators such as stage, grade, tumor size, and survival.

## Methods

### Tissue Samples

All data were anonymized and because of the retrospective nature and use of anonymized specimens and clinical data, this study was exempted by the Howard University Institutional Review Board (IRB-10-MED-24). Along with the exemption, the need for written informed consent was waived by the Howard University Institutional Review Board. We also confirm that all methods were performed in accordance with the relevant guidelines and regulations. We analyzed invasive breast ductal carcinomas (IDCs) from 202 African American women diagnosed and treated at the Howard University Hospital between 2000 and 2010. Demographic and clinical information was obtained through the Howard University Cancer Center Tumor Registry.

### Tissue Arrays

A series of tissue microarrays (TMAs) were constructed (Pantomics, Inc., Richmond, CA) consisting of 10 × 16 arrays of 1.0-mm tissue cores from well preserved, morphologically representative tumors in archived formalin-fixed, paraffin-embedded (FFPE) surgical blocks from 202 patients with primary IDCs. A precision tissue arrayer with two separate core needles for punching the donor and recipient blocks was used. The device also had a micrometer-precise coordinate system for tissue assembly on a multi-tissue block. Two separate tissue cores of IDC represented each surgical case in the TMA series. Each separate tissue core was assigned a unique TMA location number, which was subsequently linked to an Institutional Review Board-approved database containing demographic and clinical data. Using a microtome, 5-μm sections were cut from the TMA blocks and mounted onto Superfrost Plus microscope slides.

### Immunohistochemistry

Mammaglobin and GATA3 expression was assessed using immunohistochemistry (IHC), which was performed on TMA sections. Sections were stained with mouse monoclonal antibodies against GATA3 (L50-823, Biocare Medical, Concord, CA) and mammaglobin (304-1A5, Dako Agilent Technologies, SanTMA Clara, CA). IHC stained sections were scored by two independent observers (TN and AE) blinded to the clinical outcome. The sections were evaluated for the intensity of cytoplasmic (mammglobin) and nuclear (GATA3) reactivity (0–3) and the percentage of reactive cells; and an H-score was derived from the product of these measurements. Cases were categorized as having negative/weak (score <=10) or moderate/strong (score >10) expression for all three markers. The results were entered into a secure research database. Breast subtypes were defined using immunohistochemical expression of estrogen receptor (ER), progesterone receptor (PR), HER2, and Ki-67%. Luminal A was characterized by strong expression of ER or PR (H-score ≥200) and HER2 negativity. Luminal B was characterized by weaker expression of ER or PR (H-score <200) and HER2-positivity, Ki-67 > 14%, or by triple-positive expression of ER, PR, and HER2. The HER2 subtype was hormone receptor-negative with only HER2 positivity. The triple-negative subtype lacked expression of ER, PR, and HER2.

### Statistical Analysis

All immunohistochemical results were analyzed as categorical/bivariate variables (negative/weak and positive/moderate/strong) as described in the immunohistochemistry section. Clinicopathological variables analyzed for this study include ER status, PR status, HER2 status, molecular subtype, stage, grade, tumor size, overall survival and recurrence-free survival. Univariate analysis was utilized to determine the association between IHC markers and clinicopathological variables such as: ER, PR, HER2, subtype, grade, stage, and size. Chi-square c^2^ test or Fisher’s exact test, as appropriate, was used to examine the association between categorical variables. ANOVA was also utilized to compare H-scores in breast tumor subtypes. Kaplan-Meier estimates of overall survival, and disease-free survival, were plotted and a log-rank test performed to compare estimates among groups. All analyses were carried out using the SPSS 28 statistical program (SPSS Inc., Chicago, IL).

## Results

### Characteristics of the Study Population

**Figure 1:**
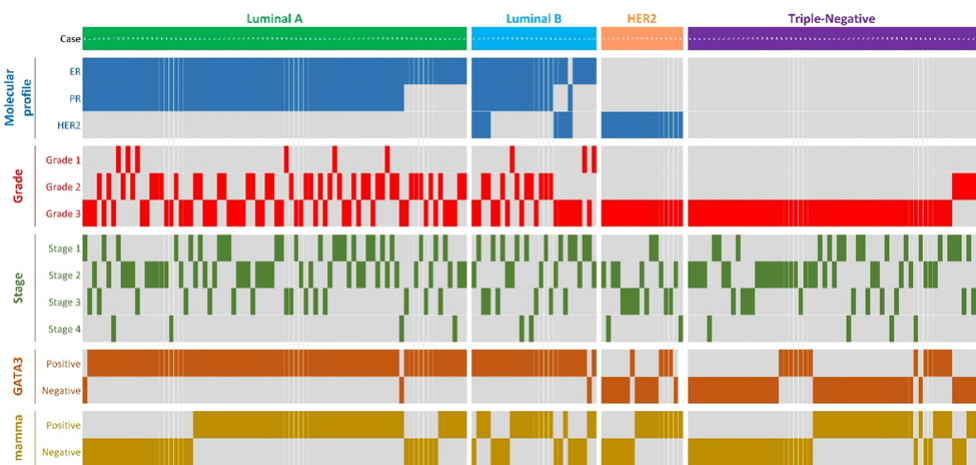
Summary of clinicopathological features, molecular profiles and IHC expression status in each patient.

Clinical and pathological characteristics of the study population are summarized in Table 1 and are presented in supplemental file 1. GATA3 and mammaglobin IHC results were available for 189 and 183 patients, respectively. Only patients with IHC results underwent further analysis. Among 189 female patients with invasive ductal carcinomas diagnosed from 2000 to 2010, the luminal A subtype was most frequent constituting 43.8% of the study population. TNBC was the second most common subtype representing 33.3% of the total number and were purposely overrepresented to improve the study of TNBC in African American women. It is noteworthy that 75% of the TNBCs demonstrated basal-like phenotype, which was determined by cytokeratin 5/6 immunohistochemistry. More than two-thirds of the tumors were stage I and II; however, the tumors tended to be of high grade, with Grade 3 tumors comprising 67.3% of the total in the study population. A summary of the patient clinicopathological features, molecular profiles and IHC expression status can be found in figure 1 which shows expression of GATA3 and mammaglobin primarily in luminal A and luminal B tumors.

### GATA3

**Figure 2:**
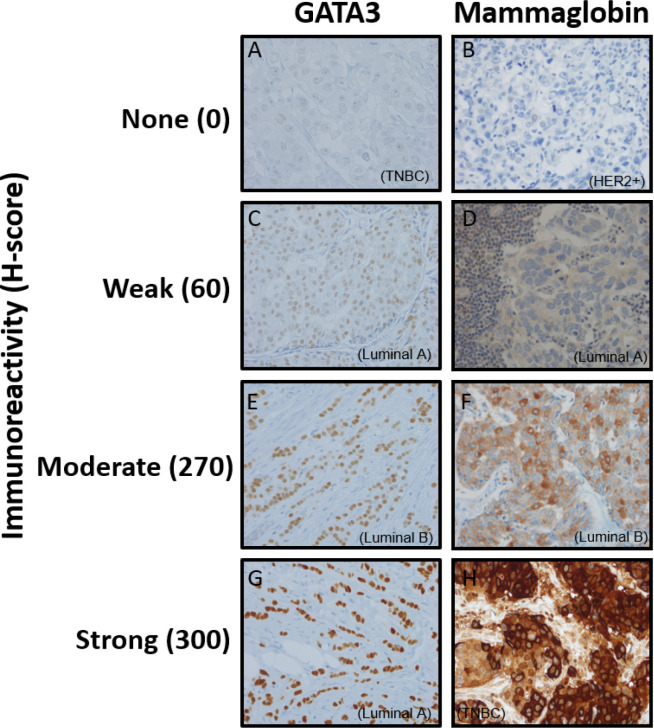
Representative images of immunohistochemical staining of GATA3 and mammaglobin.

**Figure 3:**
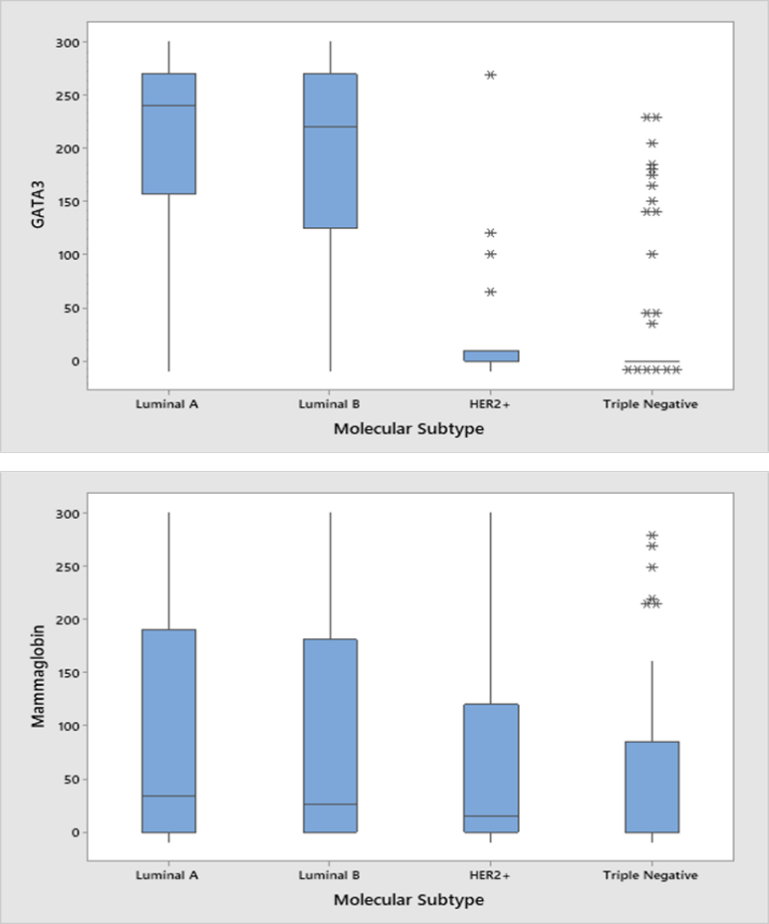
H-scores for GATA3 (A) and Mammaglobin (B) by molecular subtype.

Figure 2 demonstrates GATA3 staining intensities where 2a, 2c, 2e, and 2g demonstrate no, weak, moderate, and strong immunoreactivity. 2a shows a TNBC with no GATA3 immunoreactivity (Reactivity score=0, H-score =0). 2c shows weak GATA3 immunoreactivity (Reactivity score = 1, H-score=60) in a luminal A patient. 2e shows moderate diffuse immunoreactivity in a luminal B patient (Reactivity score = 2, H-score=270) and 2g shows strong diffuse immunoreactivity in a luminal A patient (Reactivity score = 3, H-score=300). Figure 3 demonstrates mean H-scores by subtype. GATA3 was expressed in 67.7% (128/189) of all tumors. However, GATA3 had the highest H-score expression in Luminal A (mean=218.88 ± 77.34 SD) and Luminal B (mean=212.12 ± 75.62 SD) tumors, whereas mean GATA3 H-scores were lowest in HER2 (mean=33.82 ± 71.71 SD) overexpressing and TNBCs (mean=33.20 ± 68.56 SD) (ANOVA p<0.001).

Following the dichotomization of H-scores, the frequency of positive and negative expression of GATA3 was determined for clinicopathological characteristics. GATA3 expression showed statistically significant association with a lower grade (p<0.001), ER positivity (p<0.001), or PR positivity (p<0.001) (Table 2). GATA3 was expressed in 97% and 98% and 58% of ER, PR, and HER2 positive tumors, respectively (Table 2). Among the molecular breast cancer subtypes, GATA3 was expressed in 98%, 96%, 35%, and 23% of Luminal A, Luminal B, HER2 overexpressing and TNBC subtypes, respectively, and was found to be associated with the luminal subtype (p<0.001).

**Figure 4:**
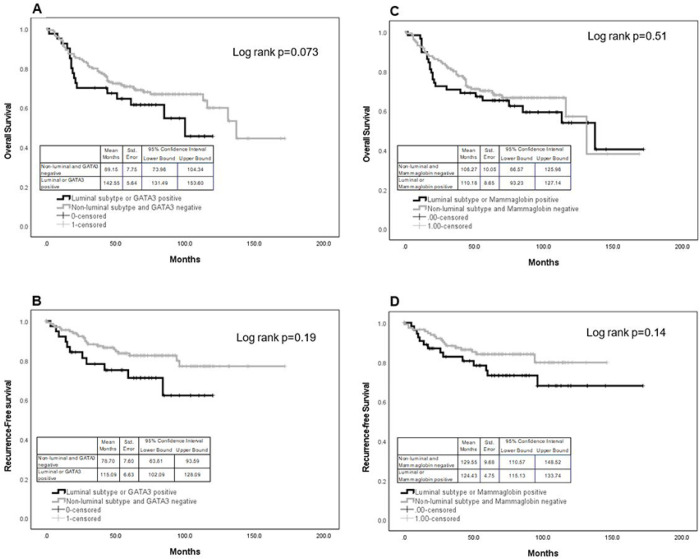
Overall and recurrence-free survival for GATA3 (A and B) and mammaglobin (C and D), respectively.

IHC expression of GATA3 was not associated with overall survival or disease-free survival (Figure 4). However, after grouping all luminal subtypes with GATA3 positive tumors and comparing them non-luminal and GATA3 negative tumors, there was a marginal significance with overall survival (4A, log rank p=0.073); mean months survival for the luminal and GATA3 positives was 142.55 months compared to 89.15 months. However, there was no association with recurrence-free survival (4B, log rank p=0.19)

### Mammaglobin

Figure 2 demonstrates mammaglobin staining intensities where 2b, 2d, 2e, and 2h demonstrate no, weak, moderate, and strong immunoreactivity. 2b shows a HER2 overexpressing subtype patients with no mammaglobin immunoreactivity (Reactivity score=0, H-score =0). 2d shows weak mammaglobin immunoreactivity (Reactivity score = 1, H-score=60) in a luminal A patient. 2f shows moderate immunoreactivity in a luminal B patient (Reactivity score = 2, H-score=270) and 2h shows strong immunoreactivity in a TNBC patient (Reactivity score = 3, H-score=300). Mammaglobin was expressed in 56.8% (104/183). Mean mammaglobin H-scores were 102.06 (±110.62 SD), 78.92 (±106.55 SD), 69.82 (± 101.71), and 53.53 (±78.40 SD) for the luminal A, luminal B, HER2+, and TNBC subtypes, respectively (ANOVA p=0.23).

Mammaglobin expression was also significantly associated with a lower grade (p=0.031), ER positivity (p=0.07), or PR positivity (p=0.022). The frequency of positive and negative expression of mammaglobin was determined for clinicopathological characteristics. Mammaglobin was expressed in 62%, 64%, and 54% of ER, PR, and HER2 positive tumors, respectively (Table 2). Positive mammaglobin expression was found in 63%, 58%, 59%, and 48% of Luminal A, Luminal B, HER2 overexpressing and TNBC subtypes, respectively, and was found to be associated with the non-TNBC subtype (p<0.043). IHC expression of mammaglobin was not associated with overall survival or disease-free survival even after grouping the luminal subtypes with mammaglobin positive tumors and comparing to non-luminals= subtypes with negative mammaglobin expression (Figure 4C, D).

### GATA3 and Mammaglobin co-expression

There was a significant correlation between GATA3 and mammaglobin expression (Pearson correlation= 0.17; p=0.022). Therefore, the sensitivity of GATA3 and mammaglobin by subtype were analyzed alone and in combination. GATA3 could detect 97% of luminal tumors and 23% of TNBCs (Table 3). However, mammaglobin had 64% sensitivity in luminal tumors. Co-expression of both markers decreased the overall detection sensitivity of luminal tumors from 97% to 86%. The expression of at least one marker was found in 43% (34/80) of TNBCs. However, co-expression of GATA3 and mammaglobin reduced the sensitivity of detecting TNBCs to 7%. In fact, 76% of TNBCs were GATA3 and mammaglobin negative.

## Discussion

The objective of this study was to characterize and evaluate the expression of GATA3 and mammaglobin in breast tumors from African American women and to determine their association with clinicopathological outcomes including breast cancer subtypes. GATA3 and mammaglobin are currently used to identify tumors of unknown primaries and metastases [1–6]. Our results confirm that GATA3 and mammaglobin demonstrate expression predominantly in luminal breast cancers, but that mammaglobin is superior to GATA3 when utilized to identify triple negative tumors in African American women, as GATA3 was found to be less frequently expressed in TNBC cases (23%) compared to mammaglobin (47%).

The high frequency of GATA3 positivity in luminal tumors aligns with its pivotal role in the differentiation of luminal progenitors to mature luminal cells[23]. Along with FOXA1 and ER-alpha, they form a hormone responsive signaling network in the normal breast that maintain epithelial differentiation by activating genes responsible for luminal features while blocking genes associated dedifferentiation or with basal or mesenchymal phenotypes [24]. GATA’s estrogen dependence greatly hinders its ability to serve as a biomarker for hormone-independent molecular breast cancer subtypes such as TNBC. Moreover, GATA3 has been found to be altered in approximately 10% of breast tumors [25]. The lack of expression may also be influenced by mutations in the gene which have been found to be overrepresented in women of African descent compared to white women with European ancestry[26]. Nakashatri *et al.* suggest that hormonal- and differentiation-signaling networks show genetic ancestry-dependent differences and it is likely that ERa:GATA3-dependent transcriptional program is more active in the normal breast of whites compared with African American women [27]. Gardner et al. [28] also showed that luminal differentiators are differentially expressed in African American women potentially contributing to more triple negative tumors.

Mammaglobin has also been previously utilized as immunohistochemical markers for identifying metastatic breast tumors, with reported overall sensitivities ranging from 50% to 87% and 10% to 79%, respectively[29]. While others have found greater sensitivities using GATA3, this study demonstrated mammaglobin’s increased ability to identify non-luminal tumors from 23% to 48%, which is much higher than previously reported by Liu *et al*.[30] (35% of ER negative tumors), Ordonez and Sahin[31] (18% of TNBCs), and Krings *et al.*[31] (26% of TNBCs). Still, given that either GATA3 or mammaglobin fail to identify more than 50% of TNBCs these markers should be supplemented with markers specific for TNBC, such as Sry-related HMG box (SOX) 10, which is found in 40%–70% of TNBCs and appears to be expressed in tumors that are negative for GATA3 [32–34]. More recently, it was demonstrated that adding SOX10 improved the sensitivity of the markers in metastatic breast cancer (sensitivity = 0.89), metastatic TNBC (0.78), and primary TNBC (0.78) [35]. Another study found that 95% of metastatic breast tumors were positive for GATA3 or SOX10 confirming SOX10’s role in identifying TNBC tumors [36].

Another interesting finding in our study is that GATA3 and mammaglobin lacked prognostic value although an association was demonstrated by others[11,37–39]. While the markers were associated with grade, there was no association with stage, tumor size (not shown), or survival. Identifying prognostic markers still remains a priority in the field of breast oncology.

The development of a TMA made up of tumors from African American women is a strength in this study. Clinical data was abstracted from the tumor registry and survival data acquired from the social security death index. KI67 IHC was also performed to differentiate the luminal A tumors from the luminal B tumors. The overrepresentation of triple negative tumors also aids in its improved understanding as African American women with TNBC continue to have worse clinical outcomes than women of European descent even after adjusting for disparities in access to health-care treatment, comorbidities and other socioeconomic factors, such as income [40,41].

The use of the TMA in this study allowed assessment of the expression of proposed diagnostic and prognostic markers and allowed for the improved characterization of tumors from African American women. It is paramount that as clinical markers are developed, that there is clinical validity and utility across groups or that the limitations are acknowledged when used in clinical practice. In conclusion, GATA3 and mammaglobin still have limited utility in detecting non-luminal tumors and should be potentially used together to identify tumors that originate in the breast.

## Figures and Tables

**Table 1.** Clinical and pathological characteristics of study population.

Parameter	Category	Frequency	%
**Age (years)**			
	<50	57	28.2
	≥50	145	71.8
**ER status (n=201)**			
	Positive	116	57.7
	Negative	85	42.3
**PR status**			
	Positive	90	47.1
	Negative	101	52.9
**HER2 status**			
	Positive	28	13.9
	Negative	173	86.1
Subtype*			
	Luminal A	88	43.8
	Luminal B	29	14.4
	Her2	17	8.5
	Triple-negative	67	33.3
**Pathologic stage (n=197)**			
	Stage 1	62	31.5
	Stage 2	82	41.6
	Stage 3	41	20.8
	Stage 4	12	6.1
**Grade**			
	Grade I	9	4.5
	Grade II	57	28.2
	Grade III	136	67.3
**Recurrence**			
	None	136	67.3
	Loco-regional	11	5.4
	Distant	21	10.4
	Never disease-free	18	8.9
	Unknown	16	7.9

**ER:** estrogen receptor; **PR:** progesterone receptor; **HER2:** Human Epidermal Growth Factor Receptor 2

**Luminal A:** ER+ or PR+, HER2−; **luminal B:** ER+ or PR+, HER2+; **triple-negative:** ER−, PR−, HER2−; **HER2+**: ER−, PR−, HER2+

**Table 2.** Summary of staining results for GATA3 and mammaglobin and their association with molecular and clinicopathological features.

		GATA3 Expression					Mammaglobin Expression				
		No or Low Expression		High Expression			No or Low Expression		High Expression		
		n=61	%	n=128	%	p value	n=79	%	n=104	%	p value
**Estrogen Receptor**											
	Negative	58	73%	21	27%		39	50%	39	50%	
	Positive	3	3%	107	97%	**<0.001**	40	38%	65	62%	0.07
**Progesterone Receptor**									104		
	Negative	59	61%	38	39%		48	50%	48	50%	
	Positive	2	2%	90	98%	**<0.001**	31	36%	56	64%	**0.022**
**HER2**											
	Negative	50	31%	113	69%		67	43%	90	57%	
	Positive	11	42%	15	58%	0.17	12	46%	14	54%	0.45
**Subtype**											
	Luminal A	2	2%	83	98%		30	38%	50	63%	
	Luminal B	1	4%	25	96%		11	42%	15	58%	
	HER2 overexpressing	11	65%	6	35%		7	41%	10	59%	
	Triple Negative	47	77%	14	23%	**<0.001**	31	52%	29	48%	0.415
**Triple Negative Status** *											
	Non-Triple Negative	12	10%	114	91%		48	40%	73	60%	
	Triple Negative	48	77%	14	23%	**<0.001**	31	51%	30	49%	0.1
**Luminal Status**											
	Non-Luminal	58	74%	20	26%		38	49%	39	51%	
	Luminal	3	3%	108	97%	**<0.001**	41	39%	65	61%	0.09
**Grade**											
	Grade I + II	5	0%	57	92%		20	32%	42	68%	
	Grade III	56	44%	71	56%	**<0.001**	61	48%	67	52%	0.031
**Stage** *											
	Stage 1	19	32%	40	68%		18	33%	36	67%	
	Stage 2	22	29%	53	71%		38	51%	37	49%	
	Stage 3	14	36%	25	64%		16	41%	23	59%	
	Stage 4	6	50%	6	50%	0.534	5	42%	7	58%	0.478

* Missing Data not included

**Table 3.** Combined expression of GATA3 and mammaglobin in luminal and triple negative breast tumors from African American women.

	Luminal Tumors			Triple negative tumors		
	+	−	Sensitivity	+	−	Sensitivity
GATA3 positive	108	3	97%	14	48	23%
Mammaglobin positive	70	40	64%	30	33	48%
GATA3 negative and mammaglobin negative	1	28	3%	22	7	76%
GATA3 positive and mammaglobin positive	63	10	86%	5	68	7%
Either GATA3 or mammaglobin positive	42	39	52%	34	46	43%

## Data Availability

The data generated and analyzed during this study are included in this published article as supplemental file 1.
